# Genome Editing for Cancer Therapy: Delivery of Cas9 Protein/sgRNA Plasmid via a Gold Nanocluster/Lipid Core–Shell Nanocarrier

**DOI:** 10.1002/advs.201700175

**Published:** 2017-09-07

**Authors:** Peng Wang, Lingmin Zhang, Yangzhouyun Xie, Nuoxin Wang, Rongbing Tang, Wenfu Zheng, Xingyu Jiang

**Affiliations:** ^1^ Beijing Engineering Research Center for BioNanotechnology CAS Key Laboratory for Biological Effects of Nanomaterials and Nanosafety CAS Center for Excellence in Nanoscience National Center for NanoScience and Technology Beijing 100190 China; ^2^ College of Materials Science and Opto‐Electronic Technology/Sino‐Danish College University of Chinese Academy of Sciences Beijing 100049 China

**Keywords:** cancer therapy, CRISPR/Cas9, genome editing, gold nanoclusters

## Abstract

The type II bacterial clustered, regularly interspaced, short palindromic repeats (CRISPR)‐Cas9 (CRISPR‐associated protein) system (CRISPR‐Cas9) is a powerful toolbox for gene‐editing, however, the nonviral delivery of CRISPR‐Cas9 to cells or tissues remains a key challenge. This paper reports a strategy to deliver Cas9 protein and single guide RNA (sgRNA) plasmid by a nanocarrier with a core of gold nanoclusters (GNs) and a shell of lipids. By modifying the GNs with HIV‐1‐transactivator of transcription peptide, the cargo (Cas9/sgRNA) can be delivered into cell nuclei. This strategy is utilized to treat melanoma by designing sgRNA targeting *Polo‐like kinase‐1* (*Plk1*) of the tumor. The nanoparticle (polyethylene glycol‐lipid/GNs/Cas9 protein/sgPlk1 plasmid, LGCP) leads to >70% down‐regulation of Plk1 protein expression of A375 cells in vitro. Moreover, the LGCP suppresses melanoma progress by 75% on mice. Thus, this strategy can deliver protein‐nucleic acid hybrid agents for gene therapy.

## 1 Introduction

CRISPR‐Cas9 system is a powerful toolbox for gene editing in various cell types and organisms.^1^ In the CRISPR‐Cas9 system, the Cas9, an endonuclease, is directed by a single guide RNA (sgRNA) to cause double‐strand break of target DNA sequences with high specificity,^2^ allowing for much greater ease of construction of knockout reagents than other methods for genetic modification.^3^, ^4^, ^5^ However, it is very challenging for the delivery of the CRISPR‐Cas9 system into cells or tissues because the plasmid encoding both Cas9 and sgRNA has strong negative charges and large size (usually exceeds 10 000 bp). Although viral vectors show high‐efficiency of gene transfection, they can result in mutagenesis, carcinogenesis, or other undesired consequences.^6^ Nonviral delivery methods such as membrane deformation^7^ or hydrodynamic injection^8^, ^9^ have been used to deliver plasmid DNA expressing Cas9 and sgRNA, but the possible damage of the target cells and/or the unsatisfactory delivery efficiency compromise the gene editing efficacy. The delivery of in vitro‐transcribed mRNA of Cas9 and sgRNA,^10^, ^11^ although promising in offering an alternative to DNA delivery, also faces challenges such as immunogenicity and the poor stability of the RNA.^12^ The delivery of the complex of Cas9 protein and sgRNA, although can realize targeting gene editing, still faces the problem of the instability of the RNA.^13^, ^14^, ^15^ The delivery of the Cas9 protein and sgRNA plasmid directly, which can not only reduce the size of the plasmid, but also avoid the instability of the RNA, is a promising strategy for CRISPR‐mediated gene editing. Furthermore, this strategy can accurately manipulate the intracellular concentration of the Cas9 protein/sgRNA plasmid complex and the editing timeframe, thus, it could potentially allow for optimization of gene editing.^13^, ^14^, ^16^, ^17^ However, the delivery of proteins also encounters difficulties: the proteins are usually not stable, and the plasmids cannot penetrate cell membrane without appropriate encapsulations.^18^ A system that can simultaneously deliver both the proteins and plasmids is highly desirable.^19^

Lipid formulations,^20^, ^21^ which are the most popular delivery systems for drug delivery at present, have the greatest potential to become routine techniques in clinical trials. The US Food and Drug Administration‐approved lipid formulations, such as Doxil, Depocyte, Daunoxome, and Ambisome have been widely used in tumor therapy. Based on their excellent features, such as high loading efficiency, good stability, and convenient preparation, the design of multifunctional lipid formulations offers great promise for drug delivery.

Gold nanocluster (GN) is a shining star in the field of nanomaterials. With a small average size (≈2 nm), excellent chemical stability, good biocompatibility, and large specific surface area,^22^ gold nanoclusters (GNs) could be used as good platforms in gene delivery.

**Scheme 1:**
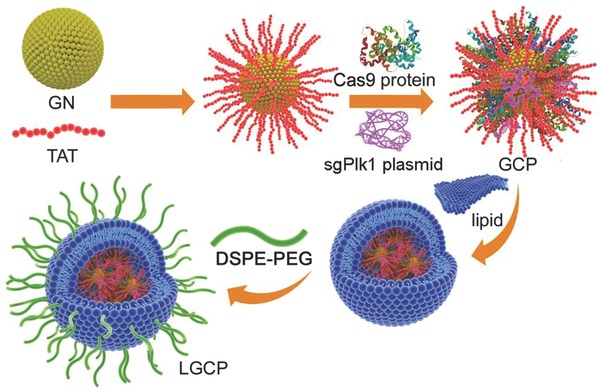
Schematic diagram of the synthesis process of the LGCP.

Herein, we report a vehicle based on lipid/GNs for delivering both Cas9 protein and sgRNA plasmid to tumor cells and tissues. We used the HIV‐1‐transactivator of transcription peptide (TAT peptide) to modify cationic GNs (TAT‐GNs), which allow improvement in nucleus‐targeting.^22^ These TAT‐GNs were synthesized through reduction of Au^3+^ in the presence of glutathione, followed by replacement of the glutathione with TAT peptide. The positively charged TAT‐GNs and the negatively charged Cas9 proteins and sgRNA plasmids were mixed to form a ternary complex (TAT‐GNs/Cas9 protein/sgRNA plasmid, we abbreviate the entire complex as “GCP”) through electrostatic interactions. Due to the excess amount of anionic sgRNA plasmids in the complex, GCP was overall negatively charged. As a core of the GCP, the TAT‐GNs can condense the complex of Cas9 proteins and sgRNA plasmids to decrease the volume of the complex to allow packaging. GCP was further encapsulated in an anionic lipid shell (1,2‐dioleoyl‐3‐trimethylammoniumpropane (DOTAP)/dioleoyl‐phosphatidylethanol‐amine (DOPE)/cholesterol, 0.8/1/0.5, n/n/n), followed by postmodification with polyethylene glycol‐phospholipids (DSPE‐PEG) on the surface of the lipid shell to form lipid‐coated GCP (this final complex we abbreviate as “LGCP”) (**Scheme** **1**). In this study, sgRNA targeting *Plk1* gene was selected because *Plk1* plays the role of master regulator in mitosis. The overexpression of *Plk1* was found in many tumor tissues and model tumor cells, e.g., A375 cells. The inhibition of *Plk1* expression can cause apoptosis of tumor cells, which provides a good strategy for tumor therapy.^23^, ^24^, ^25^ Thus, LGCP was designed to bring the powerful gene editing toolbox (Cas9 protein/sgPlk1 plasmid, here “sgPlk1 plasmid” represents the plasmid encoding only the single guide RNA, without Cas9 in this paper) into tumor cells and tissues to inhibit tumor progression with high efficiency.

## 2 Results and Discussion

### 2.1 Preparation of LGCP

**Figure 1:**
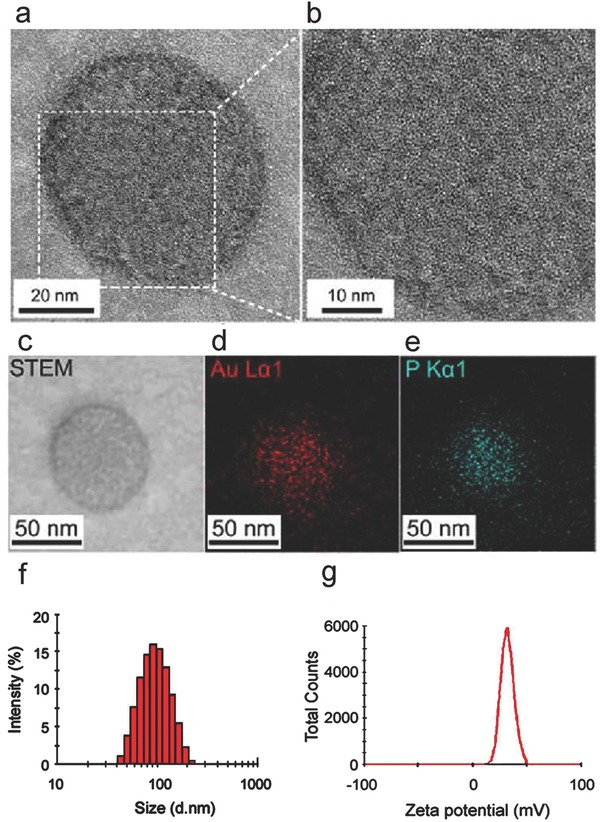
Characterizations of LGCP (polyethylene glycol‐lipid/GNs/Cas9 protein/sgPlk1 plasmid). a) HR‐TEM image of LGCP. b) Magnified image from the area marked by white dashed square in (a). c–e) TEM mapping images of LGCP. Au or P is represented by red or cyan. f) DLS analysis shows the size distribution of LGCP. g) Zeta potential of LGCP.

GNs were prepared through the glutathione (GSH) reduction method. High resolution transmission electron microscopy (HR‐TEM) indicated that the GNs were evenly dispersed (Figure S1a, Supporting Information). Dynamic light scattering (DLS) indicated that the GNs had an average diameter of ≈2.4 nm (Figure S1c, Supporting Information). The attachment of TAT peptides on the GNs did not affect their dispersity (Figure S1d, Supporting Information) but led to the increase of their sizes to ≈4.8 nm (Figure S1f, Supporting Information). The fluorescence spectrum analysis indicated that the combination of TAT peptides to the GNs resulted in a red shift of the spectrum (Figure S1b,e, Supporting Information), which was probably due to the increase of the size of the particle. The complexes of TAT‐GNs, Cas9 protein, and sgPlk1 plasmid (GCP) were encapsulated by lipid shell (DOTAP/DOPE/cholesterol) and further coated by a PEG layer (DSPE‐PEG) to form LGCP. LGCP showed well‐defined round shaped structure with an average diameter of ≈70 nm (**Figure** **1**a,b) and the particles were evenly dispersed (Figure S2c, Supporting Information). Elemental mapping showed that LGCP contains gold and phosphorus (Figure 1c–e), which represent the GNs and phospholipids (of the phosphate backbone in the sgPlk1 plasmid) respectively. DLS analysis indicated that LGCP had a hydrated diameter of about 103.7 ± 3.8 nm and a Zeta potential of 35.2 ± 5.6 mV (Figure 1f,g). By analyzing the images of the HR‐TEM and elemental mapping, we estimate that 1324 ± 172 GNs were encapsulated in a single LGCP.

Since the positive and negative charges are important parameters determining the formation of liposome structures, we optimized the ratio of lipids to TAT‐GNs. The lipids/TAT‐GNs ratio (by weight) of 1000/1 formed the expected core (TAT‐GNs/Cas9 protein/sgRNA plasmid)/shell (lipids) structure (Figures S2c and S3c, Supporting Information). By contrast, higher or lower ratios of lipids/TAT‐GNs could not form the appropriate structure (Figure S2a,b,d and Figure S3a,b,d, Supporting Information). The analysis of the fluorescence spectrum indicated that LGCP possessed an emission peak of ≈640 nm (Figure S4, Supporting Information). Compared with TAT‐GNs, there was a red shift (≈20 nm) in the spectrum of LGCP, implying the formulation of larger nanoparticles after the complexation with Cas9 protein/sgPlk1 plasmid and the encapsulation by the lipids (Figure S4, Supporting Information). Agarose gel electrophoresis analysis showed that LGCP retarded at the initial position of the gel and no free sgPlk1 plasmid appeared in the gel, while the GCP did not prevent the escape of free sgPlk1 plasmids which appeared in the gel at the same position as the control (sole sgPlk1 plasmid) (Figure S5, Supporting Information). The result of gel electrophoresis further confirmed the successful encapsulation of the GNs, Cas9 protein, and sgPlk1 plasmid by the LGCP.

The stability of LGCP in different media was characterized because the stability of transfection regents in the serum is closely related with the transfection efficiency in gene delivery, especially for the applications in vivo. LGCP was added into serum‐free Dulbecco's‐modified Eagle's medium (DMEM), Opti‐MEM reduced serum medium, or DMEM medium with 10% fetal bovine serum (FBS) (Figure S6, Supporting Information). DLS analysis indicated that LGCP incubated in serum‐free medium for 0.5 h had an average diameter of 103.7 ± 3.8 nm. When the incubation time prolonged to 72 h, the size of LGCP increased 8%, to 112.3 ± 5.8 (Figure S6, Supporting Information). When LGCP was incubated in medium containing 10% FBS for 0.5 h, the size increased to 128 ± 8.3 nm, which was only slightly larger than the size in serum‐free medium (103.7 ± 3.8 nm) at the same time point, and further incubation did not result in an apparent increase of its size (141.6 ± 10.4 nm, 72 h, 9.8% larger than the original size) (Figure S6, Supporting Information). The change of the size of LGCP in Opti‐MEM reduced serum medium showed the same tendency as those in serum‐free and 10% serum‐containing groups (Figure S6, Supporting Information). Hence, the prolonged incubation time and the high concentration of serum had no significant effect on the size of the LGCP. Thus, LGCP has the potential to have prolonged circulation time in the bloodstream and might have low cytotoxicity in vivo.

### 2.2 Confocal Microscopy Indicated Effective Cellular Uptake of LGCP

**Figure 2:**
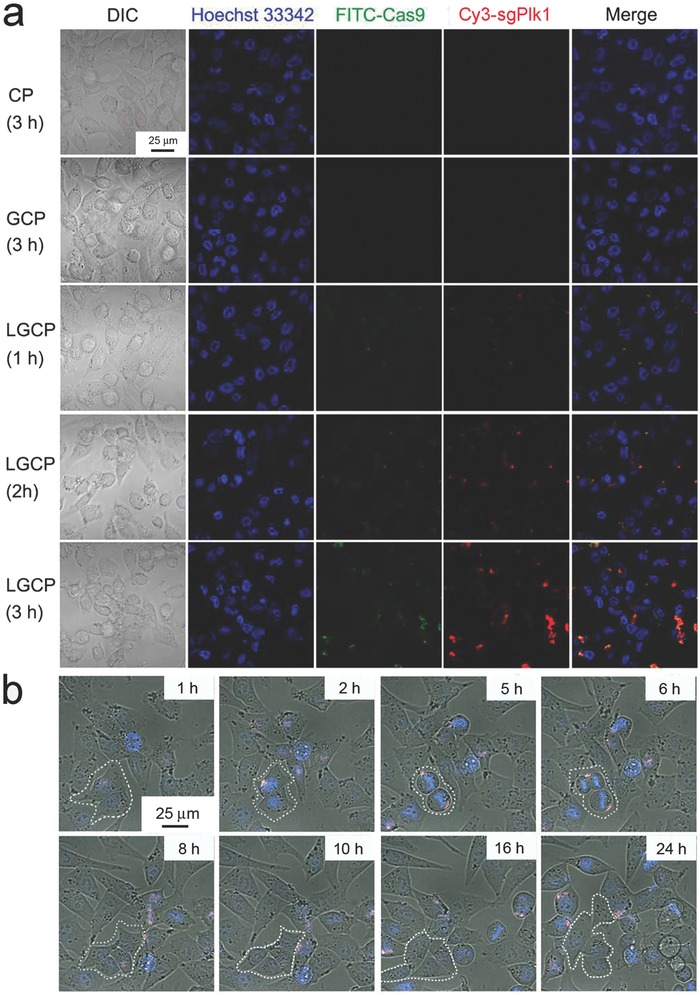
Cellular internalization of Cas9 protein/sgPlk1 plasmid formulations monitored by confocal microscopy. a) Cellular uptake of different Cas9 protein/sgPlk1 plasmid formulations. A375 cells were treated with LGCP for 1, 2, or 3 h, respectively. As controls, CP and GCP were incubated with A375 cells for 3 h. Blue: nucleus; Green: FITC‐Cas9 protein; Red: Cy3‐sgPlk1 plasmid. b) Real‐time tracing of the LGCP incubated with A375 cells for 24 h. (The white circles in the images indicate the distribution of Cas9 proteins and sgPlk1 plasmids within the cells at various time points, the cells highlighted by the white circles also display cellular division with the simultaneous separation of the internalized LGCP.) The A375 cells were incubated with LGCP for 0.5 h, stained with Hoechst 33342 for 15 min, and observed by an UltraVIEW VOX confocal system. Blue: cell nucleus; Green: FITC‐Cas9 protein; Red: Cy3‐sgPlk1 plasmid. CP, Cas9 protein/sgPlk1 plasmid; GCP, TAT‐GNs/Cas9 protein/sgPlk1 plasmid; LGCP, PEG‐lipid/TAT‐GNs/Cas9 protein/sgPlk1 plasmid.

To evaluate the ability of LGCP to enter cells, Cas9 protein and sgPlk1 plasmid were labeled with FITC (FITC‐Cas9) or Cy3 (Cy3‐sgPlk1 plasmid) respectively before the formation of Cas9 protein/sgPlk1 plasmid (CP), TAT‐GNs/Cas9 protein/sgPlk1 plasmid (GCP), and LGCP. The intracellular fluorescence of the FITC‐Cas9 and Cy3‐sgPlk1 plasmid indicated that LGCP was internalized by A375 cells only after 1 h of incubation (**Figure** **2**a). When incubation time increased to 3 h, both the fluorescence of FITC‐Cas9 protein and Cy3‐sgPlk1 plasmid were positive in almost all of the cells and the relative fluorescent intensity of FITC or Cy3 per cell at 3 h was about 8.6 and 3.9‐fold higher than those at 1 h, respectively (Figure 2a; Figure S7a, Supporting Information). By comparison, there was almost no fluorescence in the cells incubated by CP or GCP for 3 h (Figure 2a; Figure S7b, Supporting Information). The incubation of LGCP with the cells for 3 h significantly increased the intensity of the inner‐cellular fluorescence, showing about 57 (FITC) or 23 (Cy3)‐fold higher than those of the GCP (Figure S7b, Supporting Information). This result indicates that the coating of the lipid shell greatly improved the ability of the CP or GCP to enter cells. Furthermore, about 24.55% FITC‐Cas9 proteins or 21.84% Cy3‐sgPlk1 plasmids located in the regions of the nuclei, demonstrating that a large number of LGCP were in the correct subcellular location to carry out gene editing (Figure 2a; Figure S7c, Supporting Information).

Real‐time tracking of the fluorescence can illustrate the detail of the cell‐penetration and location processes of LGCP. The fluorescence of LGCP gradually appeared in the cells and aggregated to the regions of nuclei with the increase of incubation time (Figure 2b). Some LGCP aggregates separated and went into different daughter cells with the division of the mother cells (Figure 2b). These results indicate that LGCP delivered Cas9 proteins and sgPlk1 plasmids to the cells effectively. With the further increase of the incubation time, the fluorescence of FITC and Cy3 in the cells gradually became weak (Figure 2b; Figure S8, Supporting Information).

We wondered if LGCP could achieve endosomal/lysosomal escape because this process is important for efficient gene editing. After A375 cells were stained with Lysotracker Blue and incubated with LGCP, we found that the green (FITC‐Cas9 protein) and red (Cy3‐sgPlk1 plasmid) fluorescence of LGCP colocalized with blue fluorescence of Lysotracker in most of the cells. Prolonging the incubation time to about 6 h led to the separation of the green/red fluorescence from the blue fluorescence, suggesting the successful escape of the LGCP from the endosome/lysosome system (Figure S9, Supporting Information). DOTAP and TAT peptide may play important roles in the endosomal release of the gene editing complex from LGCP. On the one hand, DOTAP is a monovalent cationic lipid. Previous reports indicated that the ion‐pair effects led to efficient endosomal escape in lipid‐based formulations.^26^, ^27^ In our work, the ion‐pairs might form between the cationic DOTAP and the anionic lipids on the endosome membrane, which can induce the disassembly of LGCP and allow the release of the complex of TAT‐GNs/Cas9 protein/sgPlk1 plasmid from LGCP. Moreover, the ion‐pairs can facilitate the formation of the inverted hexagonal phase in the binding lipids, which may trigger the membrane fusion between LGCP and endosome membrane, leading to destabilization of endosome membrane.^28^ On the other hand, the cationic TAT clusters in LGCP may enhance the endosomal escape as well. Thus, LGCP facilitates the release of Cas9 protein and sgPlk1 plasmid from endosome/lysosome.

### 2.3 In Vitro Genome Editing and Inhibition of Plk1 Expression by LGCP

**Figure 3:**
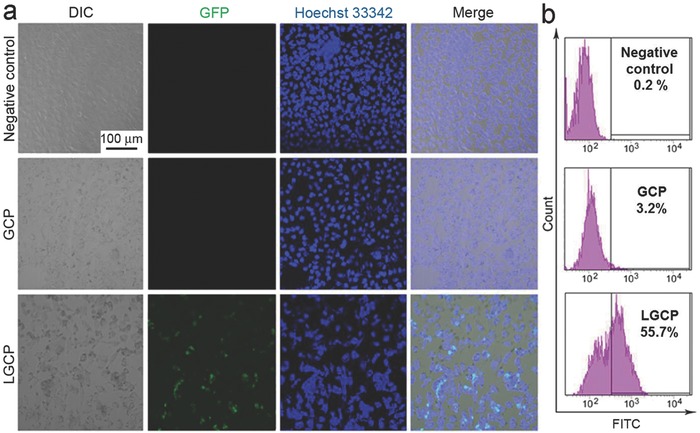
Transfection efficiency of LGCP in A375 cells. a) Confocal microscopy images show the transfection efficiency of different Cas9 protein/sgPlk1 plasmid formulations in A375 cells. b) Flow cytometry (FCM) analysis of the GFP‐positive cells. Negative control: the cells treated by PBS. GCP, TAT‐GNs/Cas9 protein/sgPlk1 plasmid; LGCP, PEG‐lipid/TAT‐GNs/Cas9 protein/sgPlk1 plasmid.

To further evaluate the transfection efficiency of LGCP, we prepared sgPlk1 plasmid encoding green fluorescent protein (GFP) (GFP‐sgPlk1 plasmid). The expression of GFP allows the convenient readout of the transfection efficiency in cells. After incubation of GCP or LGCP with A375 cells for 48 h in Opti‐MEM reduced serum medium, respectively, most of the LGCP‐treated cells became green whereas only negligible number of GCP‐treated cells became green (**Figure** **3**a). Flow cytometry analysis indicated that about 55.7% of the LGCP‐treated cells were GFP‐positive (green) (Figure 3b). Compared with LGCP, only 3.2% of the GCP‐treated A375 cells were GFP‐positive. Thus, it is evident that the coating of lipid significantly improved the transfection efficiency of Cas9 protein/sgPlk1 plasmid.

**Figure 4:**
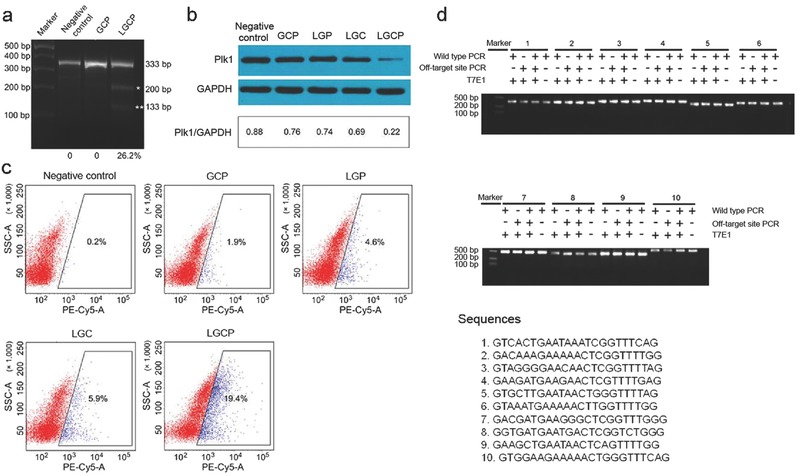
The cleavage of *Plk1* locus induced by LGCP on A375 cells. a) T7E1 assay for LGCP‐mediated indel formation. Asterisks indicate the cleaved DNA produced by T7E1 enzyme that is specific to heteroduplex DNA caused by genome editing. The mutation frequency was calculated from the proportion of cut band intensity to total band intensity ((133 bp + 200 bp)/333 bp). b) Western blot assay for analyzing the *Plk1* expression in A375 cells treated by LGCP. c) FCM analysis of apoptosis of A375 cells treated by LGCP (cells in the frame are considered as positive ones). d) T7E1 analysis of the potential off‐target effect in A375 cells induced by LGCP. Negative control, PBS; GCP, TAT‐GNs/Cas9 protein/sgPlk1 plasmid; LGP, PEG‐lipid/TAT‐GNs/sgPlk1 plasmid; LGC, PEG‐lipid/TAT‐GNs/Cas9 protein; LGCP, PEG‐lipid/TAT‐GNs/Cas9 protein/sgPlk1 plasmid.

To verify if LGCP achieve targeted cleavage of genomic DNA, we transfected A375 cells with LGCP. T7 Endonuclease I (T7E1) assay was performed to test the endogenous targeted cleavage (**Figure** **4**a) because double‐stranded breaks in genomic DNA are mostly repaired by the indel‐forming nonhomologous end joining (NHEJ) pathway without template.^29^ LGCP resulted in efficient cleavage of the target gene (*Plk1*) (26.2%) (Figure 4a). By comparison, the phosphate buffered saline (PBS) as negative control (NC) and GCP groups showed no cleavage of *Plk1* (Figure 4a). We also carried out Western blot assays to determine whether the Cas9 protein/sgPlk1 plasmid‐mediated *Plk1* gene depletion has effects on the expression of Plk1 protein. The LGCP treatment induced ≈70% down‐regulation of Plk1 protein expression compared with the NC (PBS) (Figure 4b). The control groups, such as GCP, PEG‐lipid/TAT‐GNs/sgPlk1 plasmid (LGP), or PEG‐lipid/TAT‐GNs/Cas9 protein (LGC), showed slight decrease of Plk1 expression (Figure 4b).

The apoptosis of tumor cells treated by LGCP was evaluated. Previous reports indicated that the depletion of *Plk1* might inhibit tumor cell proliferation and division and lead to tumor cell apoptosis.^23^, ^24^, ^25^ In this study, the LGCP treatment resulted in 19.4% apoptosis among A375 cells, which was about tenfold higher than that of the GCP group (1.9%). As controls, the LGP and LGC groups only induced 4.6% and 5.9% cell apoptosis, respectively (Figure 4c). Hence, the in vitro experiments demonstrated that LGCP was most effective in inhibiting tumor cell progression.

To evaluate potential off‐target effect of LGCP, Basic Local Alignment Search Tool was used to find ten potential sites matching the on‐target gene in high similarity and most likely to induce off‐target effects. T7E1 analysis indicated that A375 cells treated with LGCP showed no significant incision in ten potential off‐targeted sites (Figure 4d).

### 2.4 In Vivo Transfection Assay Indicated the Effective Tumor Inhibition

**Figure 5:**
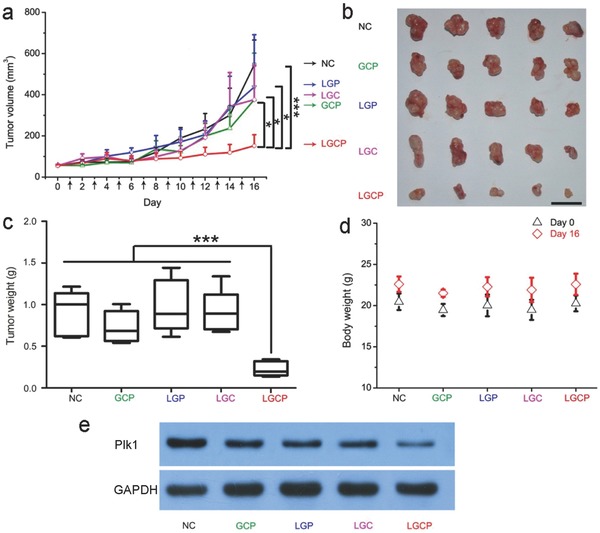
Treatment of tumor model on mice by LGCP. a) The effects of different treatments on the sizes of the tumors. The black arrows on the axis indicate the day of administrations. *, *p* < 0.05; ***, *p* < 0.001, Student' *t*‐ test. b) Photographs of the tumors excised from the mice. Scale bar: 2 cm. c) The weights of the excised tumors. d) Body weight of the mice before and after the administration of different formulations. e) Western blot analysis of the tumor tissues. NC, PBS; GCP, TAT‐GNs/Cas9 protein/sgPlk1 plasmid; LGP, PEG‐lipid/TAT‐GNs/sgPlk1 plasmid; LGC, PEG‐lipid/TAT‐GNs/Cas9 protein; LGCP, PEG‐lipid/TAT‐GNs/Cas9 protein/sgPlk1 plasmid.

The tumor inhibition effect of LGCP in vivo was evaluated on melanoma model mice. A375 cells were implanted in Balb/c nude mice subcutaneously. Intratumoral injection was performed after the volume of the tumors grew to about 50 mm^3^. The tumor development was monitored by measuring the sizes of the tumors every day for 16 d. LGCP had the most significant inhibition effect on the size of the tumor (tumor size, day 0, 55.21 ± 3.29 mm^3^; day 16, 151.39 ± 53.41 mm^3^) compared with the NC (PBS) (day 0, 56.14 ± 5.35 mm^3^; day 16, 545.18 ± 119.89 mm^3^) and other control groups. (**Figure** **5**a,b). On day 16, the average weight of the excised tumors treated by LGCP (0.23 ± 0.09 g) was significantly lower than those of the NC (0.90 ± 0.27 g), GCP (0.73 ± 0.19 g), LGP (0.98 ± 0.32 g), or LGC (0.91 ± 0.26 g)‐treated groups (Figure 5a–c). These in vivo experiments indicate that LGCP had effectively inhibited the tumor growth.

Moreover, the LGCP treatment appeared to be safe for the model mice. The body weights of the mice prior and after the LGCP administration were the same as those of the NC group, indicating a low level toxicity of LGCP (Figure 5d). Western blot assay was performed to analyze the excised tumor tissues, in the purpose of evaluating the molecular basis of the tumor inhibition by the Cas9 protein/sgPlk1 plasmid formulations. The LGCP administration significantly down‐regulated the Plk1 protein expression compared with other groups (Figure 5e), demonstrating that LGCP suppressed the tumor progression by depletion of *Plk1* gene in tumor tissues.

### 2.5 High Throughput Sequencing Confirmed the Successful Gene Editing of *Plk1* Gene

To confirm that the tumor inhibition on mice was induced by the LGCP‐mediated mutation of *Plk1* on tumor tissues, deep sequencing of *Plk1* locus of the total tumor genomic DNA was performed. The LGCP‐treated tumors showed frame shift mutations in the predicted regions of *Plk1* locus (Figure S10, Supporting Information). Frequent occurrence of nucleotide insertions or deletions (indels) appeared. This result provided the evidence that the inhibition of Plk1 protein expression was based on the disruption of the *Plk1* reading frame by LGCP‐induced indels. By comparison, there was no significant indels induced by the GCP, LGC, or LGP‐treated mice. Further experiments indicated that about 21.4% of the cells in the tumor tissues were successfully transfected after one time of injection of LGCP, which was about 20‐fold higher than that of the GCP group (Figure S11, Supporting Information). These results indicated that the successful delivery of Cas9 protein/sgPlk1 plasmid played key roles in the mutation of tumor *Plk1* gene and subsequent tumor inhibition.

In this study, we developed a general strategy for delivering Cas9 protein/sgRNA plasmid to tumor tissues. As reported, Cas9 protein shows a net theoretical charge about +22,^14^ whereas the sgRNA plasmid contains a number of phosphate groups and is highly anionic. The mixture of Cas9 protein and the sgRNA plasmid should yield a complex (CP) which is overall highly anionic. We utilized cationic GNs (+35 mV) as a core to attract and condense anionic Cas9 protein/sgPlk1 plasmid complex to form GCP, which should be anionic on its surface. The coating of cationic lipids on the surface of the anionic GCP facilitated the formation of stable lipid‐encapsulated GCP (LGCP) (Figure 1) for delivering Cas9 protein/sgPlk1 plasmid. We delivered Cas9 protein/sgPlk1 plasmid by LGCP to tumor cells and tissues to inhibit tumor growth by knocking out *Plk1* gene of the cancer cells to lead to apoptosis of cancer cells and subsequent the inhibition of the cancer progression. In vitro, we achieved up to 26.2% of Cas9‐mediated genome modification of A375 cells (Figure 4a), which was over tenfold more effective than that by traditional plasmid transfection method.^17^ The knockout of *Plk1* gene led to >70% down‐regulation of Plk1 protein expression of the A375 cells (Figure 4b). In vivo, the tumors treated by LGCP were about one fourth of the control groups in volume (Figure 5a). Thus, this approach has the potential for human tumor therapeutics. The safety of this treatment is acceptable, e.g., the body weights of the LGCP‐treated group were similar to the levels of the control group (PBS) (Figure 5d).

Transfection of Cas9/sgRNA to tumor cells and tissues in the LGCP offer a number of advantages compared with existing approaches in gene therapy. Compared with siRNA, which requires continuous administration to suppress certain genes, CRISPR/Cas9 enables genome‐level changes.^29^ Furthermore, the off‐target effect of siRNA remains a problem. By comparison, the CRISPR/Cas9 system has been reported to have a higher precision during gene editing.^30^ There was still room for us to achieve real high‐specificity cancer therapy by using the Cas9/sgRNA system. By doing this, we could greatly reduce side effects of the anti‐cancer drugs and promote the precise treatment of cancers. This strategy, compared to any other cancer treatment methods, displays advantage of treating cancers at genomic level.

## 3 Conclusion

In conclusion, we present a novel strategy for efficient delivery of Cas9 protein and sgRNA plasmid for gene therapy of cancers. By employing electrostatic interactions between the core (TAT‐GNs), gene‐editing agents (Cas9 protein/sgPlk1 plasmid), and the shell (lipid), we fabricated the vehicle for delivering Cas9 protein/sgPlk1 plasmid and achieved up to 26.2% of *Plk1* genomic modification in vitro, which was over tenfold more effective than the traditional plasmid transfection methods. The in vivo cancer *Plk1* genome‐editing induced ≈75% suppression of the melanoma progression. In the future study, by incorporating multiple sgRNAs for targeting different mutation sites in cancers, our approach will be promising for treating highly heterogeneous cancers which are extremely hard issues in clinics.

## 4 Experimental Section

*Materials*: PEG2000‐DSPE was from AVT (Shanghai, China). HAuCl_4_, GSH, TAT peptide (CYGRKKRRQRRR), cholesterol, and protamine were from Sigma‐Aldrich (St. Louis, MO, USA). DOPE and DOTAP were from AVANTI Polar Lipids (Alabaster, AL, USA). Cas9 protein and sgPlk1 plasmid were from Viewsolid Biotech (Beijing, China).

*Plasmid Construction*: sgRNA expression vector harboring a T7 promoter positioned upstream of a partial guide RNA sequence was obtained from Viewsolid Biotech (Beijing, China). To construct plasmid encoding sgRNA bearing customized 19 nt targeting sequences, a pair of compatible and annealed oligonucleotides were inserted into the backbone of the vector.

*Preparation of TAT‐Modified GNs*: TAT‐GNs were synthesized according to previous methods with a minor modification.^31^ Briefly, solutions of HAuCl_4_ (10 × 10^−3^ m, 4 mL) and GSH (50 × 10^−3^ m, 1 mL) were mixed at 25 °C. The mixture was heated to 50 °C under gentle stirring (500 rpm) to react for 24 h to form GNs. To synthesize TAT‐GNs, TAT‐peptide (50 × 10^−3^ m, 0.5 mL) was added into the solution of the GNs and the mixture was stirred for another 24 h at 25 °C to yield an aqueous solution with light‐green color.

*Preparation and Characterization of LGCP*: The core–shell nanoparticles were prepared according to the previously reported method with minor modification.^32^ In brief, cationic liposomes composed of DOTAP, DOPE, and cholesterol (0.8:1:0.5, n/n/n) were prepared through procedures of thin film hydration and ultrasonic dispersion. The negatively charged core was a ternary complex composed of TAT‐GNs, Cas9 protein, and sgPlk1 plasmid. Briefly, the solutions of TAT‐GNs and Cas9 protein were added in the solution of sgPlk1 plasmid to obtain a negatively charged ternary complex (GCP) by electrostatic interaction. The optimized weight ratio of (TAT‐GNs+Cas9 protein)/(sgPlk1 plasmid) was 2/1 (w/w). The GCP solution was kept at room temperature for 15 min, followed by encapsulation with cationic lipid shell (lipids/GCP = 1000/1, w/w) and subsequent incubation at room temperature for 15 min. Finally, the lipids/TAT‐GNs/Cas9 protein/sgPlk1 plasmid complex was further postmodified with DSPE‐PEG to yield PEG‐lipids/TAT‐GNs/Cas9 protein/sgPlk1 plasmid (LGCP). After incubation at 55 °C for 15 min, LGCP was obtained. The molar ratio of PEG in total lipids was 3%. The particle size and Zeta potential of the nanoparticles were measured by dynamic light scattering (Malvern Zetasizer nano ZS, Malvern, UK).

*TEM Characterization*: The water suspension of GNs, TAT‐GNs, or LGCP was added to carbon coated copper grids (200‐mesh) and dried in air at ambient condition. The samples were observed under TEM (Tecnai G2 20 S‐TWIN, FEI).

*Loading Capability Characterization*: To evaluate the encapsulation efficiency of the Cas9 protein and sgPlk1 plasmid, LGCP was isolated by centrifugation at 4 °C, 15 000 rpm for 30 min. The concentrations of unbound Cas9 protein and sgPlk1 plasmid in the supernatant were measured by a NanoDrop 2000 Spectrophotometer (Wilmington, Delaware USA). The encapsulation efficiency could be calculated according to the equation

(1) EE(%)=(C1−C2)/C1 × 100%

where C1 represents the original concentration of Cas9 protein or sgPlk1 plasmid in the solution and C2 represents the unbound Cas9 protein or sgPlk1 plasmid in the supernatant.

*Cas9 Protein Labeling*: Cas9 protein was labeled with FITC according to the supplier's protocol. Briefly, Cas9 protein in borate buffer was mixed with FITC solution. The final concentration ratio of the two components was ≈100 ng FITC/1 µg Cas9 protein. The mixture was incubated at 37 °C for 90 min. The unreacted FITC was removed by NAP‐5 column. The resulting FITC‐labeled protein was stored at −80 °C.

*Plasmids Labeling*: Cy3 Label IT Nucleic Acid Labeling Kit (Mirus Bio LLC. USA) was used to label the siRNA plasmid according to the supplier's protocol. Briefly, the sgPlk1 plasmids were incubated with the Label IT Reagent at 37 °C for 1 h, followed by purification of the samples with G50 Microspin Purification Column. The sample was washed with labeling buffer for twice. 0.1 volume of 5 m sodium chloride and 2 volumes of ice cooled 100% ethanol were added into the reaction solution, and kept it at −20 °C for 30 min. The labeled nucleic acids were centrifuged at 14 000 g for 30 min to remove the supernatant. 500 µL 70% ethanol (room temperature) was added and the labeled nucleic acids were centrifuged at 14 000 g for an additional 30 min. The supernatant was removed and the pellet was resuspended with sterilized water and stored at −20 °C.

*Cell Culture*: A375 cells were from the Institute of Basic Medical Science, Chinese Academy of Medical Sciences (Beijing, China). The cells were cultured in DMEM (Life Technology) supplemented with 10% FBS and 1% penicillin/streptomycin in 5% CO_2_ at 37 °C.

*Measurement of In Vitro Transfection Efficiency*: A375 cells were cultured in Petri dishes at 1 × 10^6^ cells mL^−1^ in DMEM medium containing 10% FBS and 1% penicillin/streptomycin at 37 °C in 5% CO_2_ for 24 h. The medium was replaced with Opti‐MEM reduced serum medium (Invitrogen, USA) containing PBS, GCP, LGP, LGC, or LGCP, respectively, in which the sgPlk1 plasmid or Cas9 protein was equivalent to 2 µg well^−1^, respectively. After incubation with the cells for 3 h, the medium was replaced with complete DMEM medium. The cells were incubated for another 48 h. The cells were observed under a confocal laser microscope (CLSM 710; CarlZeiss, Germany). The excitation of the GNs was set at 405 nm. CLSM 710 software was used to analyze the images.

*Real‐Time Imaging of Cells*: Cas9 protein was labeled with FITC (FITC‐Cas9) and the sgPlk1 plasmid was labeled with Cy3 (Cy3‐sgPlk1 plasmid). A375 cells were cultured in Opti‐MEM reduced serum medium containing PEG‐lipid/GNs/FITC‐Cas9 protein/Cy3‐sgPlk1 plasmid for 0.5 h, then stained by Hoechst 33342 for 15 min, and rinsed with DMEM medium. For imaging lysosome escape, the cells were stained by Lysotracker Blue (Invitrogen, USA). The cells were cultured in a chamber (Tokai Hit, 37 °C, 5% CO_2_) coupled to a spinning disk of UltraVIEW Vox confocal system (PerkinElmer, Co.). For cellular uptake process imaging, Hoechst 33342, FITC, and Cy3 were excited at 405, 488, and 561 nm respectively. For lysosome escape imaging, Lysotracker Blue, FITC, and Cy3 were excited at 405, 488, and 561 nm, respectively. The dynamic images were captured at 10 min interval for 4 h.

*T7 Endonuclease I (T7EI) Mutation Detection*: Targeted genomic loci were amplified from genomic DNA of wild type or the transfected cells. Primers to anneal about 333 bp upstream and downstream from the expected cut site were designed. The purification of the polymerase chain reaction (PCR) products is performed as described above. T7 Endonuclease I assays were performed following the protocol of the manufacturer.

Primers:

GAGTCCAGTCCTGTGCTTCC
GCTGGAATTTGGAGGAGGCT

*In Vivo Tumor Suppression*: Female Balb/c nude mice (six weeks old, 18–20 g) were from Vital River Laboratory Animal Center (Beijing, China). The experiments on animals were approved by Animal Center of Institute of Process Engineering, Chinese Academy of Sciences. The animals were raised in specific pathogen free environment.

A375 cells suspended in PBS were inoculated in mice (3 × 10^6^ cells per mouse) subcutaneously to produce melanoma models. The tumor‐laden mice were randomly divided to five groups (five mice per group) to observe the effects of PBS, GCP, LGP, LGC or LGCP treatments on tumor progression. The dose of Cas9 protein or sgPlk1 plasmid of each injection was 10 µg per mouse, respectively. The drug administrations were performed by in situ injection every other day and finished on day 15 followed by immediate euthanasia of the animals on day 16. The volumes of the tumors were calculated according to the equation: V (mm^3^) = 0.5 × length × width^2^.

*Western Bolt*: The transfected A375 cells were treated by lysis buffer (50 × 10^−3^ m HEPES, pH 7.5, 150 × 10^−3^ m NaCl, 1% Triton X‐100, 10% glycerol, 1.5 × 10^−3^ m MgCl_2_, 1 × 10^−3^ m EGTA). The concentrations of resulting proteins were quantified by BCA Protein Assay (Pierce, Rockford, IL). The lysates were separated by electrophoresis (SDS‐polyacrylamide gel) and transferred onto nitrocellulose membranes (XCell, Invitrogen). The nitrocellulose membrane was incubated with primary antibody against Plk1 (Rabbit mAb #4513, Cell Signaling Technology) at dilution of 1:1000. The protein bands were displayed by Enhanced Chemiluminescence solution. Image J software was used to analyze the protein bands.

*Sequencing of CRISPR‐Edited Plk1 Loci*: The region of genomic Plk1 was amplified by PCR using Herculase II high‐fidelity polymerase. The amplified products were purified by gel electrophoresis. 50 ng of the PCR products were used to build Libraries by Nextera protocol and sequenced by Illumina MiSeq (150 bp). The data were processed following standard Illumina sequencing procedures. In brief, reads were mapped to the PCR amplicons as reference sequences using Burrows Wheeler Aligner with custom scripts. Insertions and deletions were crosschecked against reference using VarScan2. Indel phase was calculated as the length of insertions or deletions. Indels at Plk1 existing at the same frequency across all samples, possibly arose from PCR or sequencing errors, were filtered out in final analysis. Two to five biological replicates were sequenced for in vivo tumor samples.

Primer:

F AGCCTCCTCCAAATTCCAGC
R GAGTCCAGTCCTGTGCTTCC

The first PCR and second PCR were performed for 18 cycles and 24 cycles, respectively. The products of the second PCR were gel extracted, quantified, mixed, and sequenced on a HiSeq 2500 (Illumina).

Raw FASTQ files were de‐multiplexed by FASTX‐Toolkit and processed to contain only the unique sgRNA sequence. To align the processed reads to the library, the designed sgRNA sequences from the library were assembled into a Burrows–Wheeler index using the Bowtie build‐index function. The Bowtie aligner was used to align the reads to the index. After the alignment, the number of uniquely aligned reads for each library sequence was calculated. The numbers of reads for each unique sgRNA for a given sample were normalized as follows: Normalized reads per sgRNA = (reads per sgRNA/total reads for all sgRNAs in sample) × 10^6^ + 1.

## Conflict of Interest

The authors declare no conflict of interest.

## Supporting information

Supplementary
