# Supplementary material for: Genome Editing for Cancer Therapy: Delivery of Cas9 Protein/sgRNA Plasmid via a Gold Nanocluster/Lipid Core–Shell Nanocarrier
Source: Adv Sci (Weinh). 2017 Sep 7;4(11):1700175. doi: 10.1002/advs.201700175 (PMC5700650; doi:10.1002/advs.201700175)
Supplement: Supplementary file 1 — Supplementary [file ADVS-4-na-s001.pdf]

Copyright WILEY-VCH Verlag GmbH & Co. KGaA, 69469 Weinheim, Germany, 2017.

ADVANCED SCIENCE
Open Access

Supporting Information

for *Adv. Sci.,* DOI: 10.1002/advs.201700175

Genome Editing for Cancer Therapy: Delivery of Cas9 Protein/sgRNA Plasmid via a Gold Nanocluster/Lipid Core–Shell Nanocarrier

Peng Wang, Lingmin Zhang, Yangzhouyun Xie, Nuoxin Wang, Rongbing Tang, Wenfu Zheng, and Xingyu Jiang**

Supporting Information

Genome editing for cancer therapy: Delivery of Cas9 protein/sgRNA plasmid via a gold nanocluster/lipid core-shell nanocarrier

Peng Wang, Lingmin Zhang, Yangzhouyun Xie, Nuoxin Wang, Rongbing Tang, Wenfu Zheng, and Xingyu Jiang**

** Corresponding authors: xingyujiang@nanoctr.cn (Xingyu Jiang), zhengwf@nanoctr.cn (Wenfu Zheng)*

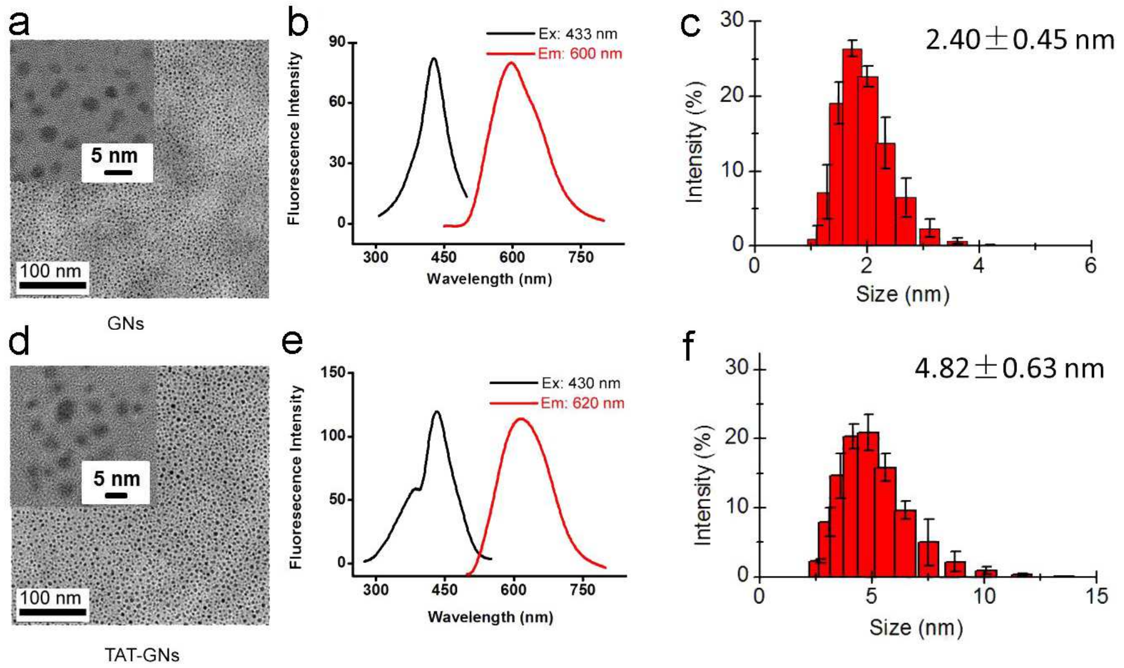

Figure S1 HR-TEM images, fluorescent spectra, and size distribution of the GNs and TAT-GNs. a) TEM image of the GNs. b) Fluorescence spectra of the GNs. c) DLS results show the size distribution of the GNs. d) TEM image of the TAT-GNs. e) Fluorescence spectra of the TAT-GNs. f) DLS results show the size distribution of the TAT-GNs. Ex, excitation spectrum; Em, emission spectrum.

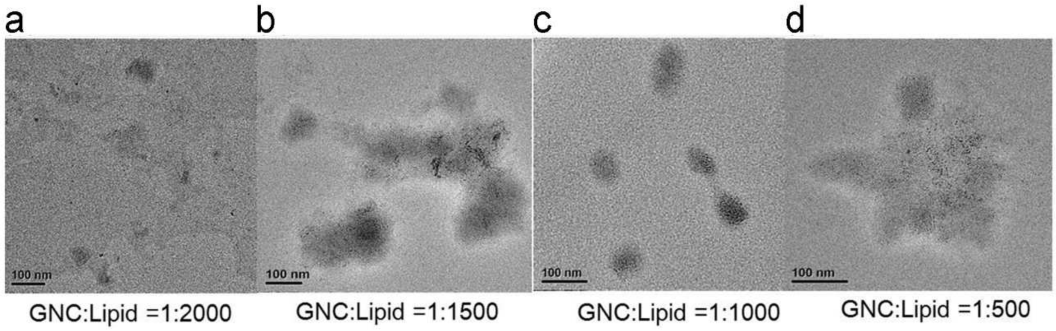

Figure S2 HR-TEM images of LGCP with different lipid/TAT-GNs weight ratios. a) 2000:1. b) 1500:1. c) 1000:1. d) 500:1.

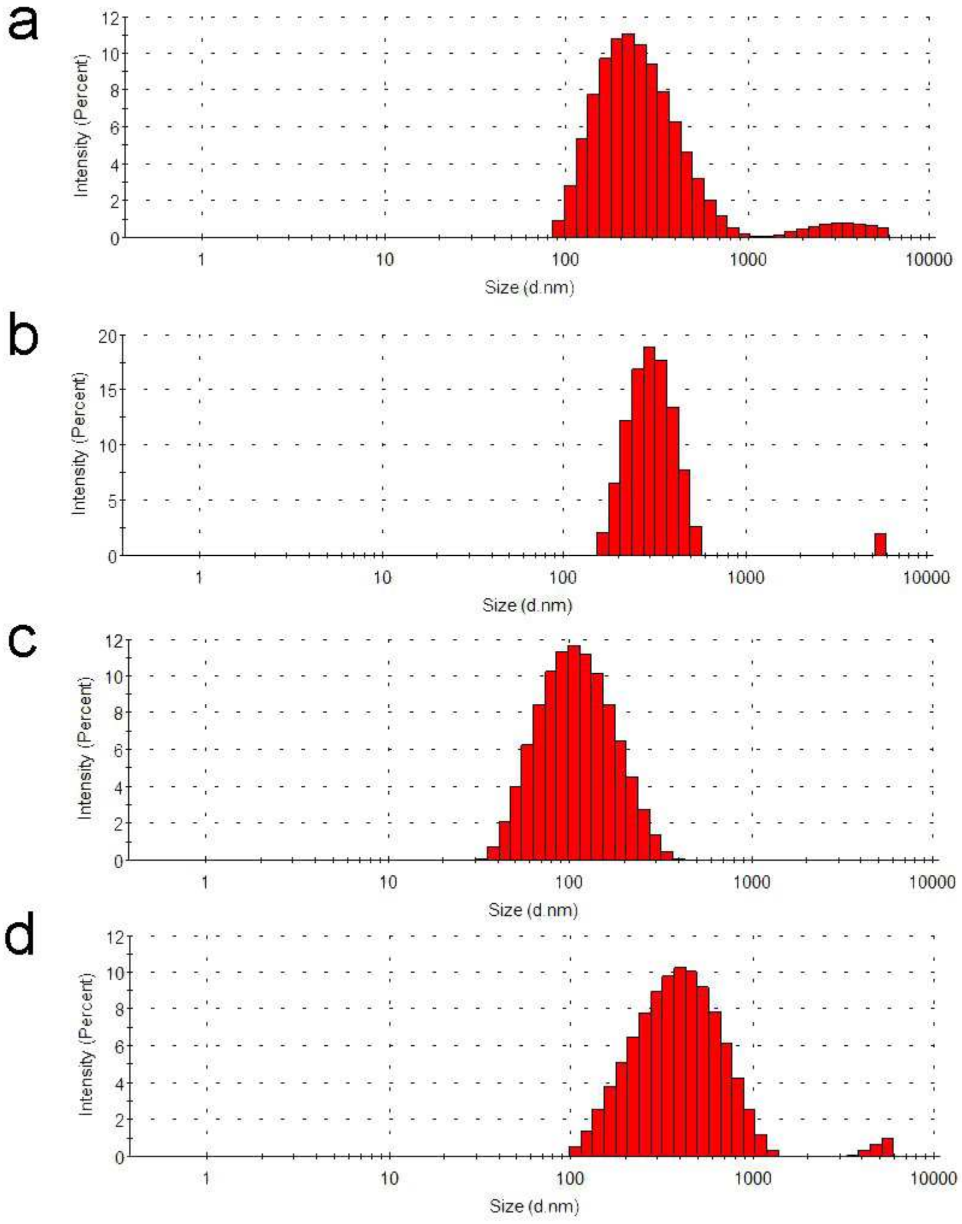

Figure S3 DLS of LGCP with different lipid/TAT-GNs weight ratios. a) 2000:1. b) 1500:1. c) 1000:1. d) 500:1.

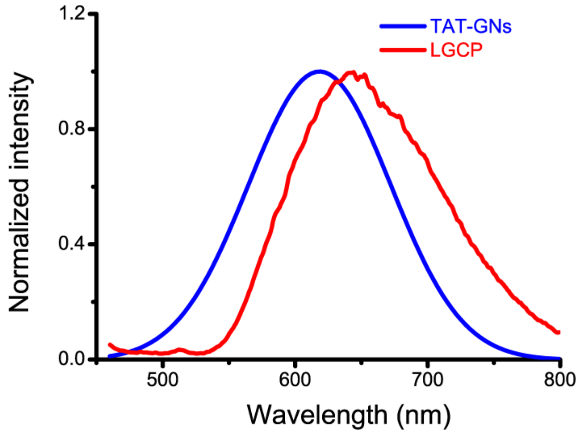

Figure S4 Fluorescence emission spectra of the TAT-GNs and LGCP. The TAT-GNs and LGCP were excited at 405 nm.

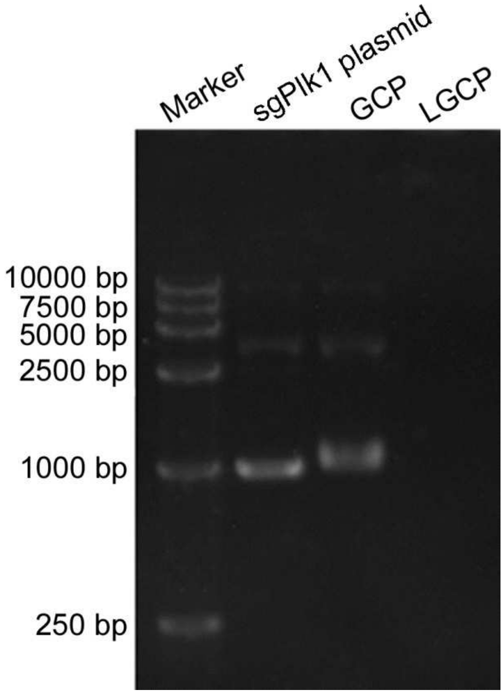

Figure S5 Gel electrophoresis analysis of the Cas9 protein/sgPlk1 plasmid formulations.

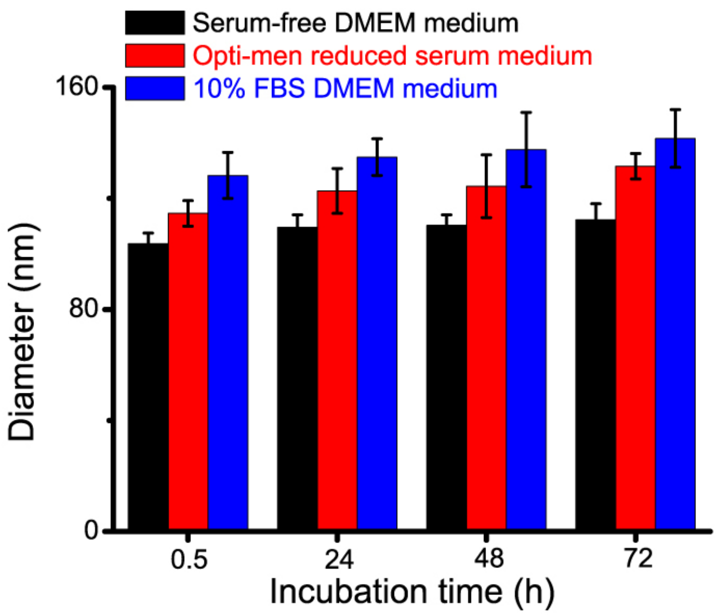

Figure S6 The diameters of the LGCP in different media conditions

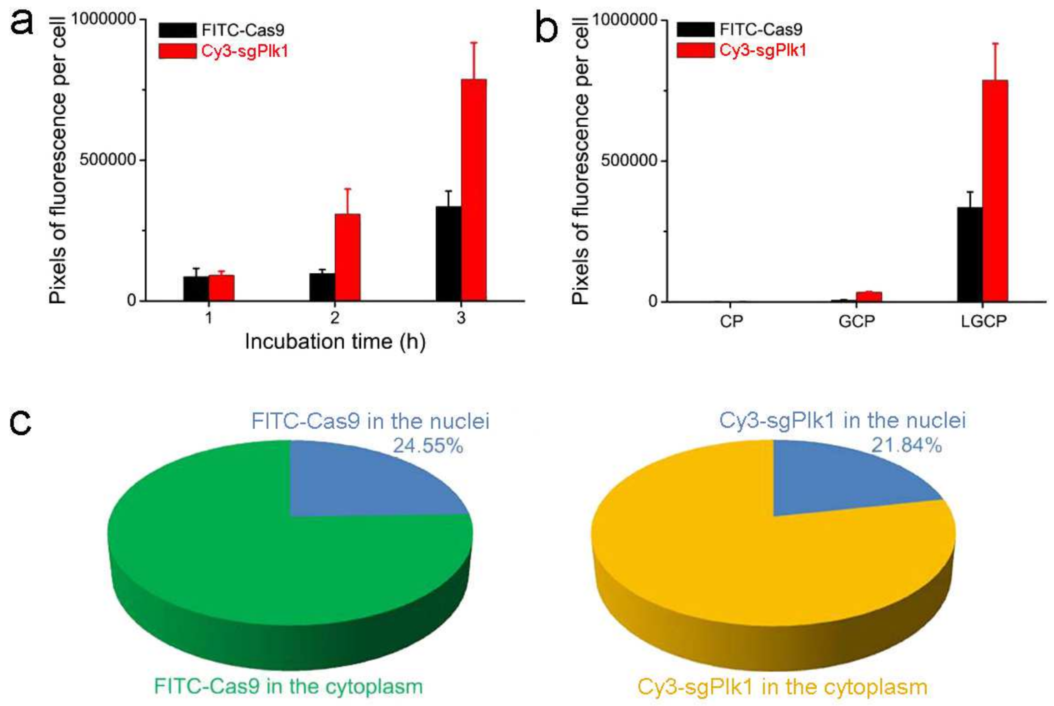

Figure S7 The intensity of fluorescence in A375 cells after the co-delivery of FITC-Cas9 and Cy3-sgPlk1 by LGCP. a) The relative intensity of fluorescent of FITC and Cy3 per cell at different incubation time points. The cells were incubated with LGCP

for 1, 2, and 3 h, respectively. b) The relative intensity of fluorescence of FITC and Cy3 in the cells treated by different Cas9 protein/sgPlk1 plasmid formulations after incubation for 3 h; c) The ratio of FITC-Cas9 protein or Cy3-sgPlk1plasmid in the cytoplasm and nuclei. The intensity of fluorescence was counted by Image J software in the CLSM images.

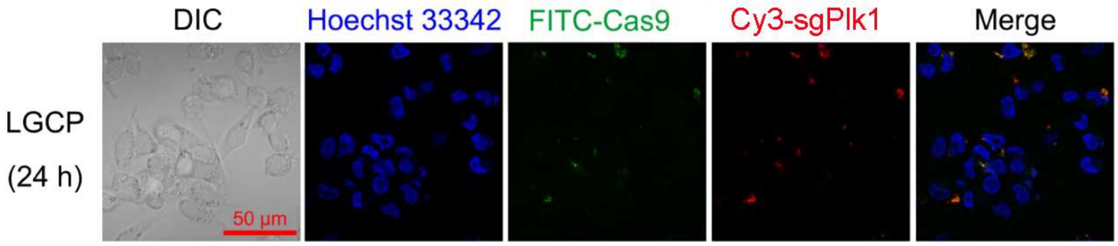

Figure S8 Cellular internalization of FITC-Cas9 protein/Cy3-sgPlk1 plasmid delivered by the LGCP. A375 cells are incubated with the LGCP for 24 h.

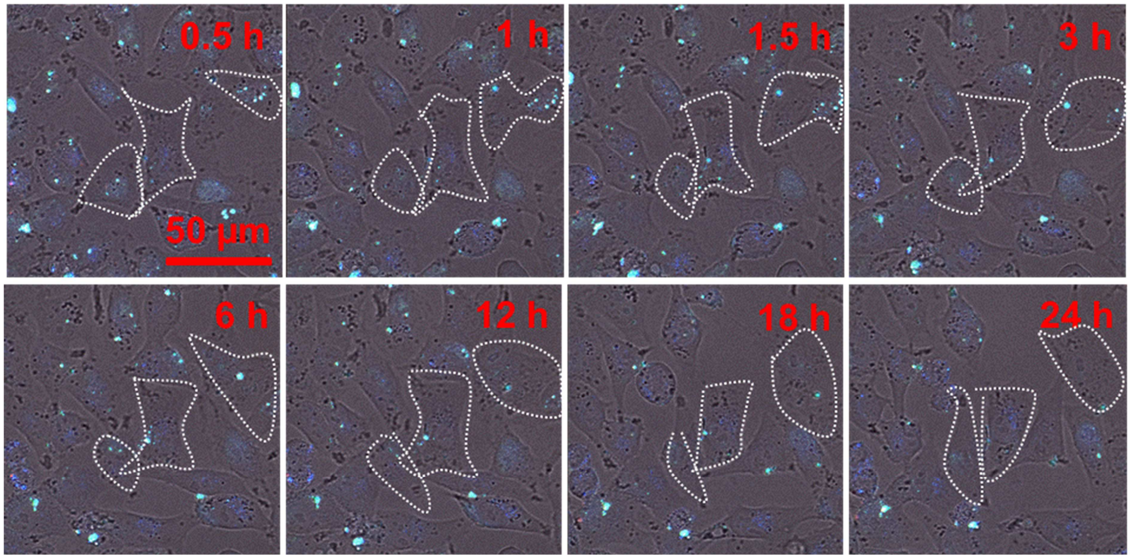

Figure S9 Real time monitoring of the lysosome escape of the Cas9 protein/sgPlk1plasmid. The Cas9 protein was labeled by FITC (FITC-Cas9) and the sgPlk1 plasmid was labeled by Cy3 (Cy3-sgPlk1). The A375 cells were incubated with 100 nM Lysotracker Blue for 2 h, followed by incubation with the LGCP for 1 h. The cells were observed with an UltraVIEW VOX Confocal system.

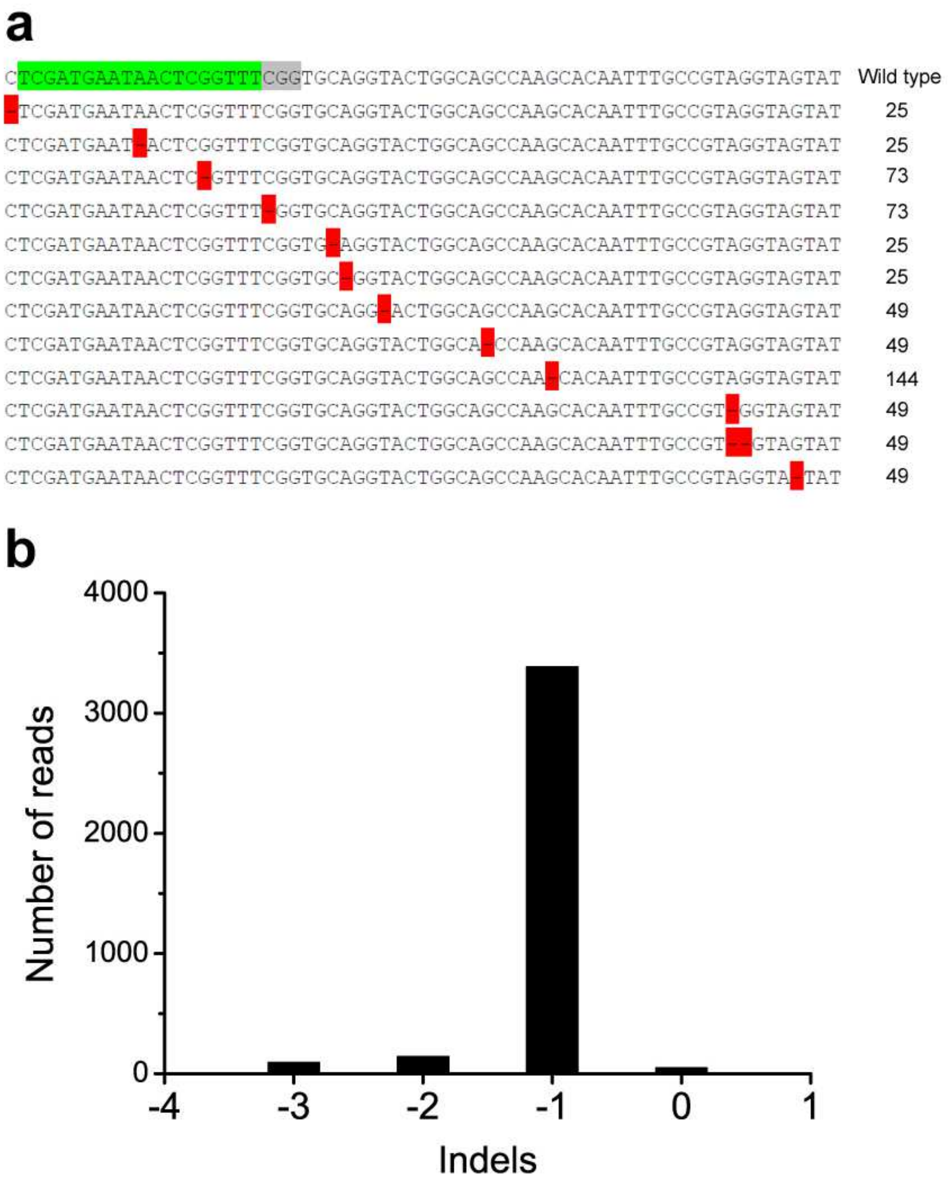

Figure S10 Targeted indel mutations induced by LGCP. a) DNA sequencing analysis of mutated alleles in the targeted Plk1 locus. Alleles shown were amplified from pooled genomic DNA isolated from the tumors in 5 mice. The wild-type sequence is shown at the top with the target site highlighted in green, and the PAM sequence is highlighted in grey. The mutation sites are marked with red. b) Histograms of on-target indel sizes for Plk1. Each subpanel shows the distribution of indel sizes for a single sgRNA at its intended genomic target locus.

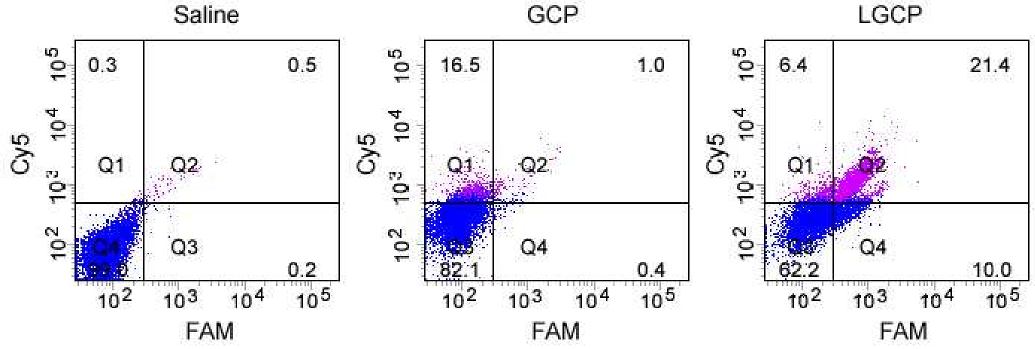

Figure S11 FCM analysis of the cells in the tumor tissue after 1 time injection of LGCP.
